# Nephrotoxicity in patients with solid tumors treated with anti-PD-1/PD-L1 monoclonal antibodies: a systematic review and meta-analysis

**DOI:** 10.1007/s10637-020-01039-5

**Published:** 2021-01-06

**Authors:** Han Li, Jinsheng Xu, Yaling Bai, Shenglei Zhang, Meijuan Cheng, Jingjing Jin

**Affiliations:** Department of Nephrology, Hebei Key Laboratory of Vascular Calcification in Kidney Disease, Hebei Clinical Research Center for Chronic Kidney Disease, The Fourth Hospital of Hebei Medical University, 12 Jiankang Road, Shijiazhuang, 050011 People’s Republic of China

**Keywords:** Anti-PD-1/PD-L1 monoclonal antibodies, Chemotherapy, Meta-analysis, Solid tumors, Nephrotoxicity

## Abstract

*Background* Programmed death-1 (PD-1) and programmed death ligand 1 (PD-L1) have dramatically improved cancer therapy for many patients. Adverse kidney effects have been found to be an important complication but have unclear mechanisms. *Methods* We searched Embase, PubMed, and the Cochrane Library to identify potential eligible studies. All included studies were randomized controlled trials (RCTs) examining patients with solid tumors treated with anti-PD-1/PD-L1 monoclonal antibodies (mAbs) and/or chemotherapy. The relative risk (RR) was used to assess the risk of nephrotoxic events. *Results* We included 27 clinical trials (15,063 patients). Compared with chemotherapy, the RR of all-grade nephritis was significantly increased with anti-PD-1/PD-L1 mAbs (RR = 2.77, 95% CI: 1.09–6.99, *P* = 0.03). Furthermore, anti-PD-1/PD-L1 mAbs plus chemotherapy can significantly increase the RR of all-grade nephritis (RR = 2.99, 95% CI: 1.07–8.35, *P* = 0.04). There was also a significant increase in the RRs of all-grade increased blood creatinine (RR = 1.88, 95% CI: 1.24–2.86, *P* = 0.003) and acute kidney injury (AKI) (RR =3.35, 95% CI: 1.48–7.60, *P* = 0.004). *Conclusions* Anti-PD-1/PD-L1 mAbs can significantly increase nephrotoxicity in patients with solid tumors, especially when combined with chemotherapy. During the application of these drugs, we should remain aware of nephrotoxicity for better efficacy. *Trial registration number and date of registration* Not applicable.

**Supplementary Information:**

The online version contains supplementary material available at 10.1007/s10637-020-01039-5.

## Introduction

According to estimates from the World Health Organization in 2015, cancer is the first or second leading cause of death before the age of 70 years in 91 of 172 countries, and it ranks third or fourth in an additional 22 countries [1]. Immune checkpoint inhibitors (ICIs) are the most recent breakthroughs in the treatment of cancer, and these agents have dramatically increased the therapeutic options for multiple cancers. Since December 2015, the Food and Drug Administration has approved two anti-PD-1 mAbs (nivolumab and pembrolizumab) and three anti-PD-L1 mAbs (atezolizumab, durvalumab and avelumab). They function by increasing the activity of the immune system to inhibit the inactivation of T lymphocytes and thereby enhance anticancer and cytotoxic effects [2]. Compared with previous standards of care, cancer immunotherapy has led to significant improvements for patients in terms of survival and quality of life [3].

However, immune system activation is detrimental not only to the survival of cancer cells but also to certain types of healthy tissues [4]. Thus, a new group of adverse events, called immune-related adverse events (irAEs), has been recognized. Renal irAEs are rare, with an estimated incidence of 2% with anti-PD-1/PD-L1 mAbs and 5% with combination therapy in a review of published phase 2 and 3 trials, but more recent studies have suggested that the incidence of AKI is higher than that initially reported [5, 6]. Acute interstitial nephritis (AIN) is the most commonly reported pathology, and other forms of nephrotoxicity mostly manifest as increased blood creatinine and AKI [7]. One recent meta-analysis [8] examined the risk of nephrotoxicity associated with anti-PD-1/PD-L1 mAbs using control groups that received placebo, chemotherapy or other immunotherapy but did not include the latest research. In contrast, our meta-analysis focused on nephrotoxicity in patients who received anti-PD-1/PD-L1 mAbs alone or in combination with chemotherapy. The combination therapy of anti-PD-1/PD-L1 agents and chemotherapy has become increasingly prevalent, but the effect of this more aggressive treatment on the risk and severity of nephrotoxicity relative to chemotherapy alone remains unknown.

To investigate the relationship between the incidence risk of immune-related nephrotoxic events and anti-PD-1/PD-L1 mAbs, we performed this meta-analysis. Our systematic review and meta-analysis investigated the RR of nephrotoxicity in patients with solid tumors treated with anti-PD-1/PD-L1 mAbs alone, anti-PD-1/PD-L1 mAbs plus chemotherapy, or standard chemotherapy alone.

## Methods

We searched Embase, PubMed, and the Cochrane Library to identify eligible studies. All included studies were RCTs that examined patients with solid tumors who received anti-PD-1/PD-L1 mAbs and/or chemotherapy. Combined RRs and 95% confidence intervals (CIs) and fixed- or random-effects methods were used to evaluate the nephrotoxicity caused by anti-PD-1/PD-L1 mAbs during treatment. This systematic review and meta-analysis was conducted according to the guidelines of the Cochrane Handbook for Systematic Reviews of Interventions [9], and the results were reported according to the PRISMA statement [10].

### Search strategy

The PubMed, Cochrane and Embase databases were searched for RCTs using the following key words: “PD-1”, “PD-L1”, “nivolumab”, “pembrolizumab”, “atezolizumab”, “durvalumab”, and “avelumab” for publications on or before June 25, 2020.

### Inclusion and exclusion criteria

According to our analysis design, the inclusion criteria were as follows: (1) studies on humans with solid tumors; (2) prospective RCTs; (3) studies that compared a PD-1/PD-L1 inhibitor with chemotherapy or a PD-1/PD-L1 inhibitor plus chemotherapy with the same chemotherapy agent (with or without placebo); and (4) studies that directly compared the nephrotoxicity data of patients receiving and not receiving anti-PD-1/PD-L1 mAbs treatment (nephritis, increased blood creatinine and AKI).

Studies were excluded if they were phase I trials, single-arm studies, or trials where patients had no adverse renal events in either treatment arm; if patients received other agents simultaneously, such as targeted drugs; if they were retrospective studies, meeting abstracts, case reports, unfinished studies, duplicate reports, letters, or reviews; and if they were in any language other than English.

### Data extraction

Two authors (J.J. and H.L.) independently evaluated all studies for eligibility by initially checking the titles, abstracts, and full texts of the studies following the patient, intervention, comparison, and outcome (PICO) chart [11]. The following information was extracted from all eligible studies: first author’s name, year of publication, trial phase, treatment groups, primary endpoint, underlying solid malignancy, number of patients in each group, chemotherapy agents, and adverse events (AEs). The two categories of AEs were all-grade (1 to 5) and high-grade (3 to 5) renal AEs, namely, increased blood creatinine, AKI, and nephritis.

### Data analysis

The risk of bias was assessed using Review Manager 5.3 software. Two authors independently assessed the quality of the included RCTs using the Cochrane risk of bias tool [12]. The relative risk (RR) was used to assess the risk of nephrotoxic events. We used Stata (version 12.0) to evaluate publication bias.

Heterogeneity among the RCTs was quantified using the Q test and I^2^ statistics. If the I^2^ value was less than 50%, a fixed-effects model was used [13, 14]; otherwise, a random-effects model was used. Sensitivity analysis was performed by removing one study at a time and recalculating the results. All *P* values were 2-tailed, and a P value below 0.05 was considered significant.

## Results

### Literature search

**Fig. 1:**
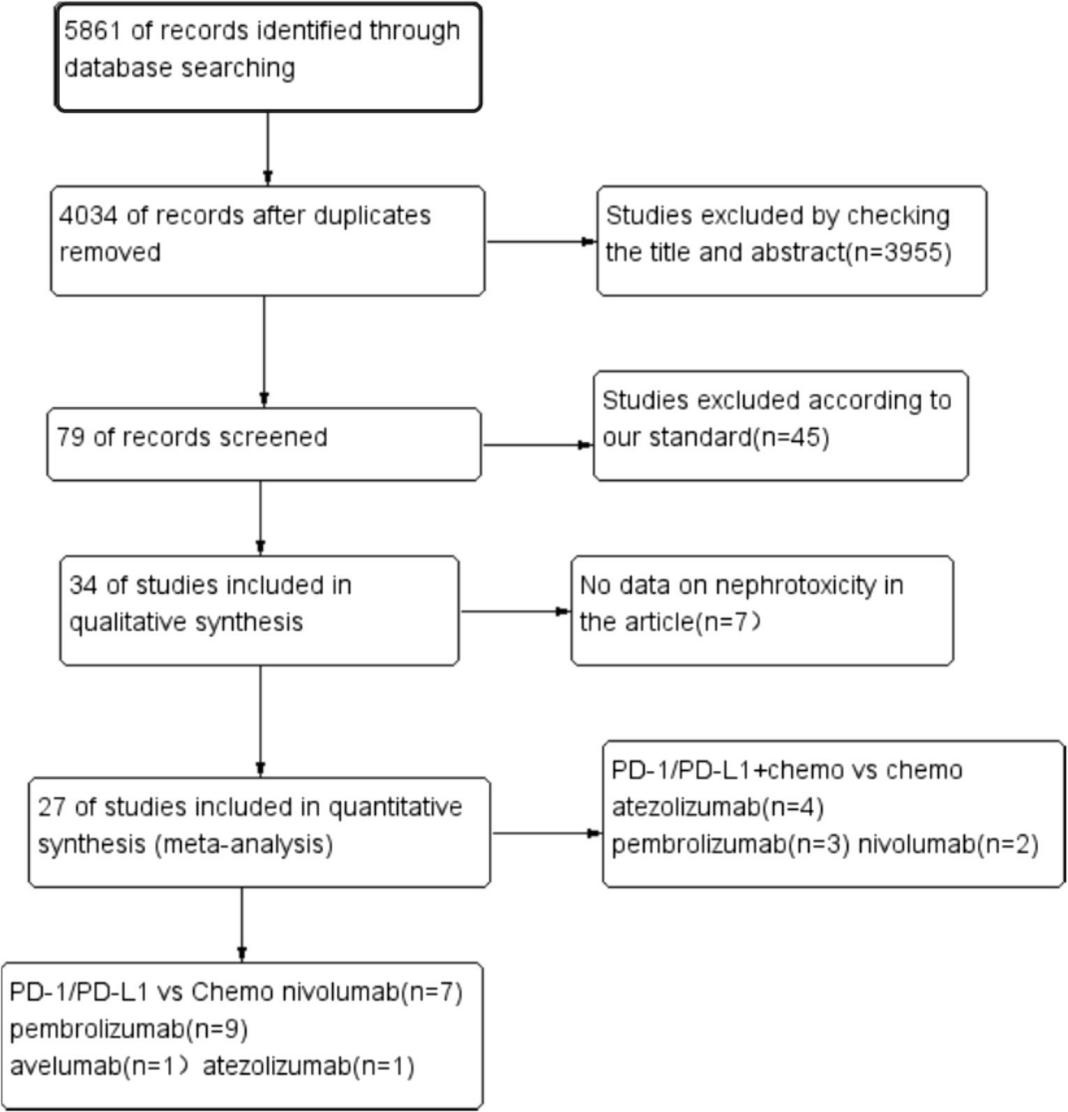
Flowchart depicting the RCT selection process

Our initial search yielded 5861 potentially relevant clinical trials. After the removal of overlapping studies from the three databases and a review of the titles and abstracts, we initially excluded 5827 studies because they did not fulfill our criteria. The excluded studies included review articles, retrospective studies, case reports, phase I trials, single-arm studies, nonrandomized clinical trials, and studies of non-solid tumors. After a review of the full texts of the remaining 34 studies, we excluded 7 trials because they had no information related to nephrotoxicity (Fig. 1). The 27 eligible studies examined patients with non-small cell lung cancer (NSCLC, *n* = 13), melanoma (*n* = 8), carcinoma of the head and neck (*n* = 2), renal cell carcinoma, small cell lung cancer, urothelial carcinoma, and breast cancer (1 each). None of the included RCTs examined durvalumab. The 27 studies in this meta-analysis examined 15,063 patients. Depending on the tumor type, the standard treatment the patient receives may be chemotherapy or ipilimumab, and four articles included RCTs of combination therapy or comparisons with ipilimumab.

Eighteen of the 27 studies examined anti-PD-1/PD-L1 mAbs vs. chemotherapy alone, and the other 9 studies examined anti-PD-1/PD-L1 mAbs plus chemotherapy vs. chemotherapy alone. The 18 studies of anti-PD-1/PD-L1 mAbs vs. chemotherapy examined patients treated with nivolumab (7 studies, 2536 patients), pembrolizumab (9 studies, 6452 patients), atezolizumab (1 study, 101 patients), and avelumab (1 study, 758 patients) (Reference: [15–32]). The 9 studies of anti-PD-1/PD-L1 mAbs plus chemotherapy vs. chemotherapy examined patients treated with atezolizumab (4 studies, 2776 patients), pembrolizumab (3 studies, 1286 patients), and nivolumab (2 studies, 1154 patients) (Reference: [33–41]). The literature was distributed from 2014 to 2019.

Tables 1 and 2 show the baseline details and the relevant all-grade and high-grade renal AEs in each trial. In this analysis, we graded all laboratory values according to the National Cancer Institute Common Terminology Criteria for Adverse Events version 4.0.

**Table 1:** Characteristics of the 18 randomized controlled trials that compared anti-PD-1/PD-L1 monoclonal antibodies vs. chemotherapy

Year	Trial phase	Tumor type	Treat arms	pts	Nephritis		Blood creatinine		AKI	
					G1–5	G3–5	G1–5	G3–5	G1–5	G3–5
2014	3	Melanoma	Niv 3 mg/kg every 2 weeks	206	N	N	1	0	1	1
			Dac 1 g/m^2^ every 3 weeks	205	N	N	1	0	0	0
2015	2	Melanoma	Pem 2 mg/kg every 3 weeks	178	1	0	N	N	N	N
			Pem 10 mg/kg every 3 weeks	179	1	0	N	N	N	N
			ICC every 3 weeks	171	0	0	N	N	N	N
2015	3	Melanoma	Pem10mg/kg every 2/3 weeks	555	1	0	N	N	N	N
			Ipilimumab 3 mg/kg every 3 weeks	256	1	1	N	N	N	N
2015	3	NSCL	Niv 3 mg/kg every 2 weeks	287	N	N	5	0	1	0
			Doc 75 mg/m^2^ every 3 weeks	268	N	N	1	0	0	0
2015	3	Melanoma	Niv 3 mg/kg every 2 weeks	268	N	N	2	0	N	N
			ICC every 3 weeks	102	N	N	0	0	N	N
2016	3	NSCL	Pem 200 mg every 3 weeks	154	1	1	3	0	N	N
			platinum-based chemotherapy	150	0	0	15	1	N	N
2016	3	Head-and-neck	Niv 3 mg/kg every 2 weeks	236	N	N	N	N	1	0
			Methotrexate/Doc/cetuximab	111	N	N	N	N	2	1
2016	2/3	NSCL	Pem 2 mg/kg every 3 weeks	339	N	N	6	0	N	N
			Pem 10 mg/kg every 3 weeks	343	N	N	7	0	N	N
			Doc 75 mg/m^2^ every3weeks	309	N	N	0	0	N	N
2017	3	Urothelial Carcinoma	Pem 200 mg every 3 weeks	266	2	2	13	2	15	7
			ICC every 3 weeks	255	0	0	15	1	7	3
2017	3	NSCL	Niv 3 mg/kg every 2 weeks	267	N	N	5	1	N	N
			Platinum-based ICC every 3 weeks	263	N	N	16	0	N	N
2017	3	SC-NSCL	Niv3mg/kg every 2 weeks	131	1	1	4	0	0	0
			Docetaxe l75 mg/m^2^ every 3 weeks	129	0	0	2	0	1	1
2017	3	Melanoma	Pembrolizumab 10 mg/kg every 3 weeks	277	2	2	N	N	N	N
			ipilimumab 3 mg/kg every 3 weeks	278	0	0	N	N	N	N
2018	3	Head-and-neck	Pem 200 mg every 3 weeks	246	N	N	0	0	1	1
			Methotrexate/Doc	234	N	N	2	0	1	0
2018	3	NSCL	Avelumab 10 mg/kg every 2 weeks	393	N	N	N	N	1	1
			Docetaxel 75 mg/m^2^ every 3 weeks	365	N	N	N	N	0	0
2018	3	Melanoma	Pembrolizumab 200 mg every3wees	509	2	2	N	N	N	N
			placebo every 3 weeks	502	1	0	N	N	N	N
2018	3	NSCL	Atezolizumab	56	1	1	N	N	N	N
			Docetaxel	45	0	0	N	N	N	N
2019	3	NSCL	Pem 200 mg every 3 weeks	636	3	1	N	N	N	N
			Platinum-based ICC every 3 weeks	615	0	0	N	N	N	N
2019 3		Renal cell carcinoma	Niv 3 mg/kg every 3 weeks	37	N	N	1	0	N	N
			Everolimus 10 mg every day	26	N	N	4	0	N	N

*Niv* nivolumab, *ICC* investigator’s choice of chemotherapy, *N* not available, *NSCLC* non-small cell lung cancer, *Doc* docetaxel, *Dac* dacarbazine, *PFS* progression-free survival, *Pem* pembrolizumab, *Pac* Paclitaxel, *UC* urothelial cancer, *Ave* avelumab

**Table 2:** Characteristics of the 9 randomized controlled trials that compared anti-PD-1/PD-L1 monoclonal antibodies plus chemotherapy vs. chemotherapy

Year	Trial phase	Tumor type	Treat arms	pts	Nephritis		Blood creatinine		AKI	
					G1–5	G3–5	G1–5	G3–5	G1–5	G3–5
2016	3	Advanced melanoma	Nivolumab 1 mg/kg + ipilimumab 3 mg/kg	94	N	N	2	1	N	N
			Ipilimumab 3 mg/kg + placebo	46	N	N	0	0	N	N
2017	3	Advanced melanoma	Niv 1 mg/kg + ipilimumab 3 mg/kg every 3 weeks	313	N	N	14	1	N	N
			Ipilimumab 3 mg/kg+ placebo	311	N	N	5	0	N	N
2018	3	Non-squamous NSCLC	Pem 200 mg + Platinum-based ICC every 3 weeks	405	7	6	36	2	21	8
			Placebo + Platinum-based ICC every 3 weeks	202	0	0	11	0	1	1
2018	3	ES-SCLC	Ate 1200 mg + Chemo (Car + Eto) every 3 weeks	198	1	1	1	0	4	2
			Placebo + Chemo (Car + Eto) every 3 weeks	196	1	0	0	0	1	0
2018	3	Squamous-cell NSCLC	Pem 200 mg + Chemo (Car+[Nb-]pac) every 3 weeks	278	2	2	N	N	0	0
			Placebo + Chemo (Car+[Nb-]pac) every 3 weeks	280	2	2	N	N	1	1
2018	3	First-line NSCLC	Ate 1200 mg + Bev + Car + Pac every 3 weeks	393	3	1	N	N	2	1
			Bev 15 mg/kg + Car + Pac every 3 weeks	394	0	0	N	N	1	1
2018	3	Triple-negative BC	Ate 840 mg + Nb-pac100 mg/m2 d1,8,15 every4weeks	452	1	0	N	N	N	N
			Placebo+ Nb-pac100 mg/m2 d 1,8,15 every4weeks	438	0	0	N	N	N	N
2016	2	Non-squamous NSCLC	Pem 200 mg + Chemo (Car + pemetrexed) every 3 weeks	59	N	N	6	0	2	2
			Chemo (Car +pemetrexed) every 3 weeks	62	N	N	4	0	1	0
2019	3	Non-squamous NSCLC	Ate 1200 mg + CarAUC6 q3w + Nb-pac100mg/m2qw	473	4	1	26	2	9	4
			CarAUC6q3w + Nb-pac100mg/m2qw	232	0	0	7	0	3	1

*Chemo* chemotherapy, *Car* carboplatin, *ES-SCLC* extensive-stage small-cell lung cancer, *Niv* nivolumab, *Ate* Atezolizumab, *Eto* etoposide, *BC* breast cancer, *Bev* bevacizumab, *AUC* area under the curve

### Nephrotoxicity: Anti-PD-1/PD-L1 mAbs vs. chemotherapy

#### All- and high-grade increased blood creatinine and AKI

The anti-PD-1/PD-L1 mAbs and chemotherapy groups had no significant differences in RR for all-grade increased blood creatinine and AKI and no significant differences for high-grade increased blood creatinine and AKI (Fig. S1 and Table S1).

#### All- and high-grade nephritis

**Fig. 2:**
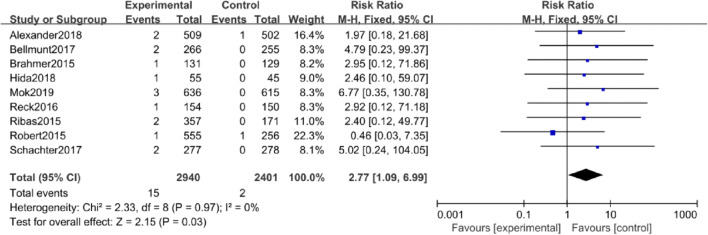
Forest plot for all-grade nephritis in studies that compared anti-PD-1/PD-L1 mAbs and chemotherapy

When comparing anti-PD-1/PD-L1 mAbs vs. chemotherapy, there was a significant increase in the RR of all-grade nephritis (RR =2.77, 95% CI: 1.09–6.99, *P* = 0.03; Fig. 2).

#### Nephrotoxicity: anti-PD-1/PD-L1 mAbs plus chemotherapy vs. chemotherapy

##### All- and high-grade increased blood creatinine and AKI

**Fig. 3:**
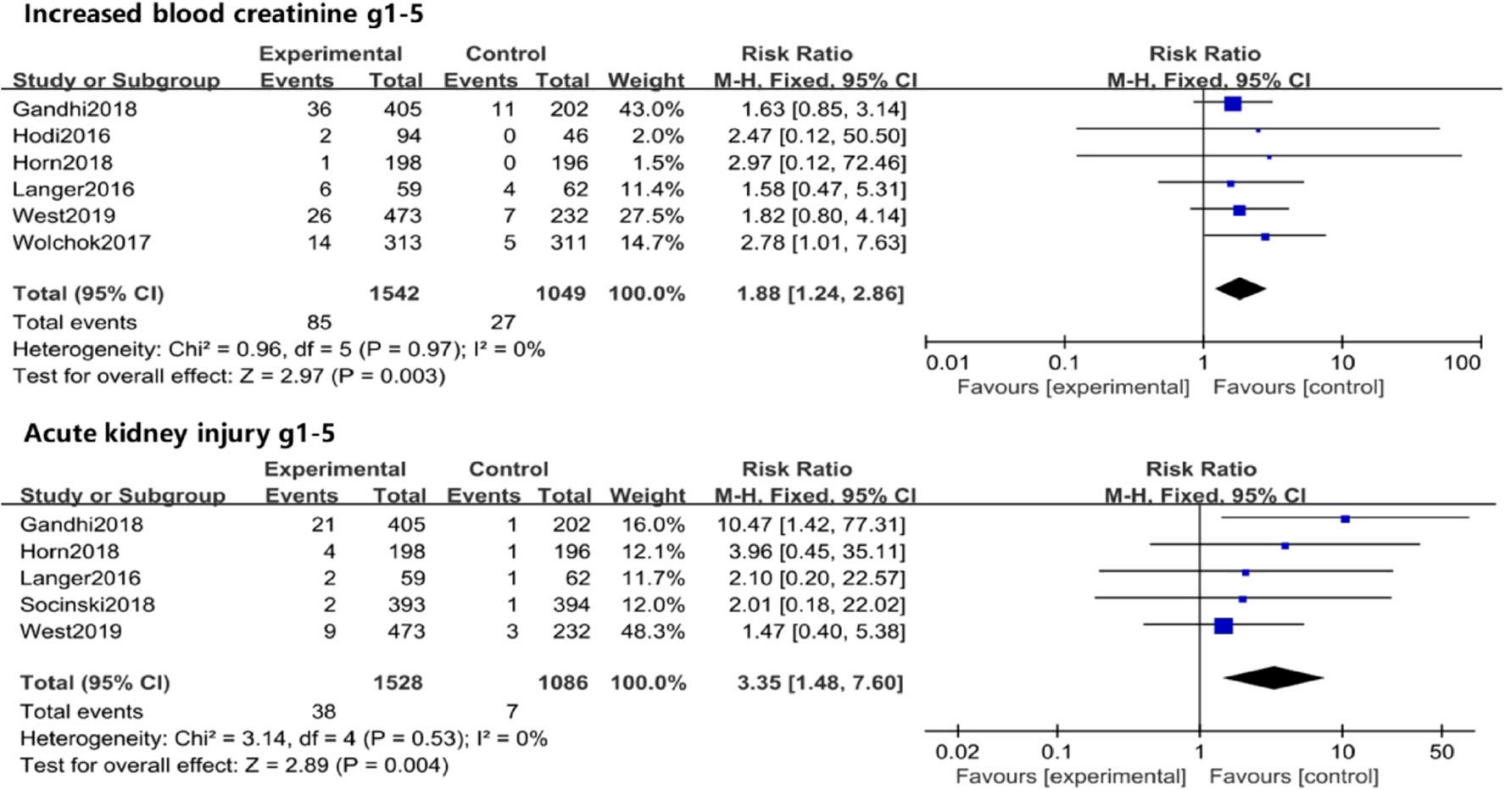
Forest plot for all grade increased blood creatinine and acute kidney injury caused by anti-PD-1/PD-L1 mAbs plus chemotherapy

When comparing anti-PD-1/PD-L1 mAbs plus chemotherapy and chemotherapy, there was a significant increase in the RR of all-grade increased blood creatinine (RR =1.88, 95% CI: 1.24–2.86, *P* = 0.003) and AKI (RR =3.35, 95% CI: 1.48–7.60, *P* = 0.004; Fig. 3). The two groups had no significant differences in the RRs of high-grade increased blood creatinine and high-grade AKI (Fig. S2 and Table S2).

##### All- and high-grade nephritis

**Fig. 4:**
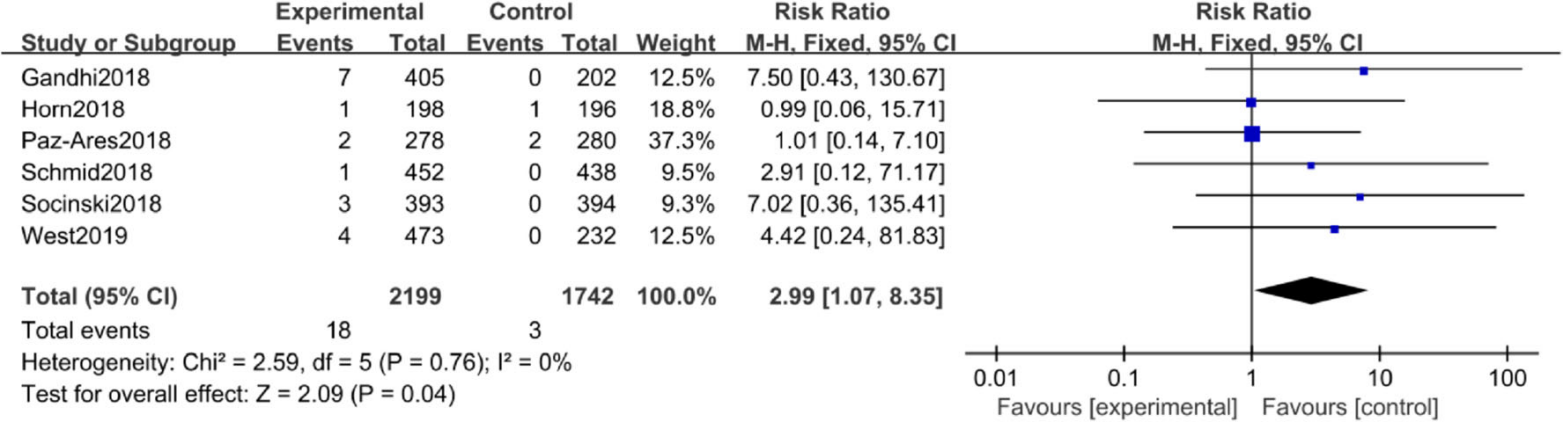
Forest plot for all-grade nephritis in studies that compared anti-PD-1/PD-L1 mAbs plus chemotherapy and chemotherapy

When comparing anti-PD-1/PD-L1 mAbs plus chemotherapy and chemotherapy, there was a significant increase in the RR of all-grade nephritis (RR =2.99, 95% CI: 1.07–8.35, *P* = 0.04; Fig. 4).

### Quality assessment and publication bias

**Fig. 5:**
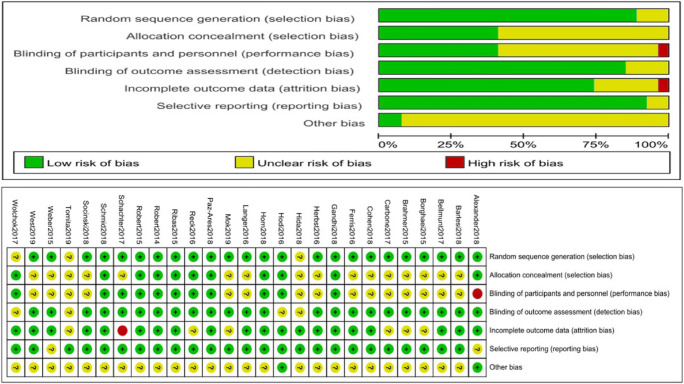
Risk of bias summary. **a** Bar chart comparing the percentage of the risk of bias for each included RCT. Low risk of bias (green), high risk of bias (red), and unclear risk of bias (yellow). **b** Risk of bias for each included RCT, representing low risk of bias (+), high risk of bias (−), and unclear risk of bias (?)

All studies were randomized controlled trials. Analysis using the Cochrane risk of bias tool indicated a low risk of bias for all included studies (Fig. 5). We used a fixed effects model for most comparisons due to the low heterogeneity among the included studies. Only one comparison used a random effects model and sensitivity analysis, and the results were not affected. The results of Begg’s test and Egger’s test indicated no evidence of publication bias.

## Discussion

The results of our analysis of 27 clinical trials including 15,063 cancer patients indicated that anti-PD-1/PD-L1 mAbs were associated with a higher risk of all-grade nephrotoxicity than control treatments. The 18 studies that examined anti-PD-1/PD-L1 mAbs vs. chemotherapy alone were distributed from 2014 to 2019, and the 9 studies that examined anti-PD-1/PD-L1 mAbs plus chemotherapy vs. chemotherapy alone were distributed from 2016 to 2019. Anti-PD-1/PD-L1 mAbs monotherapy was applied early, and the studies had a wider time distribution. Combination strategies with conventional and immunotherapies were approved later but have gradually become the focus of attention. Similar to the literature we retrieved, the related RCTs were mostly distributed in the last 4 years. Our findings bridged the gap in previous studies by comparing the risk of renal toxicity in cancer patients receiving anti-PD-1/PD-L1 mAbs with or without chemotherapy vs. chemotherapy alone.

In our results, there was a significant increase in the RR of all-grade nephritis in patients receiving anti-PD-1/PD-L1 mAbs alone. Previous studies have shown that acute tubulointerstitial nephritis (ATIN) is the most commonly reported pathological lesion in patients who have received anti-PD-1/PD-L1 mAbs therapy [42]. Based on a recent retrospective cohort analysis of more than 1000 people, the majority of potential immune checkpoint inhibitor-related events were secondary to varying degrees of tubular and interstitial inflammation and injury [43]. These results are consistent with our results. From the perspective of nephrotoxicity, acute interstitial nephritis (AIN) is the most common biopsy-proven diagnosis in patients treated with checkpoint inhibitors who develop kidney injury. The mechanism of renal irAEs is still a research focus. According to theories supported by most researchers, T cells are more likely to lose tolerance to native kidney antigens in the presence of anti-PD-1/PD-L1 mAbs, and uninhibited T cells may activate the typical drug-induced hypersensitivity reaction pathway more vigorously [44]. Thus, AIN is induced by anti-PD-1/PD-L1 mAbs, which may be due to the reprogramming of the immune system, leading to the loss of tolerance against endogenous kidney antigens [44]. The interpretation of pathological types is of great help in the treatment of immune-related nephrotoxicity. When treating patients with anti-PD-1/PD-L1 mAbs alone, physicians should be alert to the possible clinical manifestations of nephritis. If there are potential alternative reasons for AKI, a lower threshold to perform a renal biopsy should be used.

When comparing anti-PD-1/PD-L1 mAbs plus chemotherapy and chemotherapy, there was also a significant increase in the RRs of all-grade increased blood creatinine and AKI. Compared with monotherapy, anti-PD-1/PD-L1 mAbs plus chemotherapy led to increased nephrotoxicity, which can cause AKI. In a combined analysis of 3695 patients treated with ICIs, AKI was more common in patients receiving combination therapy than in patients receiving ICI monotherapy [5], which is consistent with our results. The combined literature revealed the following characteristics of ICI-related AKI. Most patients had AKI with tubulointerstitial presentation, including normal urinary output, granular casts, aseptic leukocyturia, and low-grade (0.1 g per day) or no proteinuria [43]. A review of 13 cases from seven academic centers in the USA concluded that the great majority of cases of AKI with ICIs are due to AIN [5]. According to our analysis, AKI was likely to be secondary to varying degrees of tubular and interstitial inflammation and injury. However, it is important to note that there have been recent reports of glomerular diseases, including lupus nephritis, vasculitis, and podocytopathies [45]. In addition, chemotherapy drugs can also cause many types of kidney injury, such as AIN, thrombotic microangiopathy, and acute tubular necrosis, which may aggravate damage to the tubule and interstitium and lead to higher nephrotoxicity. Treatment should be suspended for complications above grade 2, and a corticosteroid regimen should be used promptly for complications above grade 3 [46]. Therefore, when using anti-PD-1/PD-L1 mAbs plus chemotherapy, we should be more vigilant about nephrotoxicity and changes in blood creatinine. Continuous laboratory testing and necessary treatment are the basis of our treatment process.

This meta-analysis has some limitations. First, due to the lack of reports of renal adverse events in many studies, the number of qualified publications is limited. In addition, due to the heterogeneity of the selected RCTs and different diagnostic criteria, the identification of immune-related nephrotoxicity by researchers and organizations may not be completely consistent. Second, we were unable to obtain the patient’s personal information or the patient’s long-term follow-up data, so we did not consider the details of immune-related nephrotoxicity. Similarly, although patients with advanced tumors sometimes develop kidney metastases, the lack of patient-level information limits our ability to identify specific abnormalities in patients with renal adverse events.

## Conclusion

Anti-PD-1/PD-L1 mAbs can significantly increase nephrotoxicity in patients with solid tumors, especially when combined with chemotherapy. During the application of these drugs, we should remain aware of nephrotoxicity for better efficacy.

## Supplementary Information

ESM 1 (PDF 335 kb)
