# Supplementary material for: Nephrotoxicity in patients with solid tumors treated with anti-PD-1/PD-L1 monoclonal antibodies: a systematic review and meta-analysis
Source: Invest New Drugs. 2021 Jan 6;39(3):860–70. doi: 10.1007/s10637-020-01039-5 (PMC8068624; doi:10.1007/s10637-020-01039-5)
Supplement: Supplementary file 1 — (PDF 335 kb) [file 10637_2020_1039_MOESM1_ESM.pdf]

Table S2 The risk estimates that compared PD-1/PD-L1 inhibitors plus chemotherapy vs. chemotherapy

AE type	RR (95%CL), P value	
	Grade1-5	Grade3-5
Increased blood creatinine	1.88(1.24-2.86),0.003	2.31(0.49,10.85),0.29
AKI	3.35(1.48,7.60),0.004	3.00(1.01,8.94),0.05
Nephritis	2.99(1.07,8.35),0.04	2.38(0.74,7.68),0.15

Increased blood creatinine g1-5

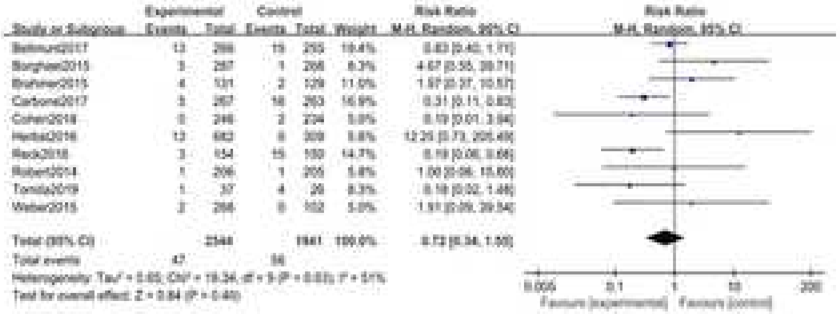

Increased blood creatinine g3-5

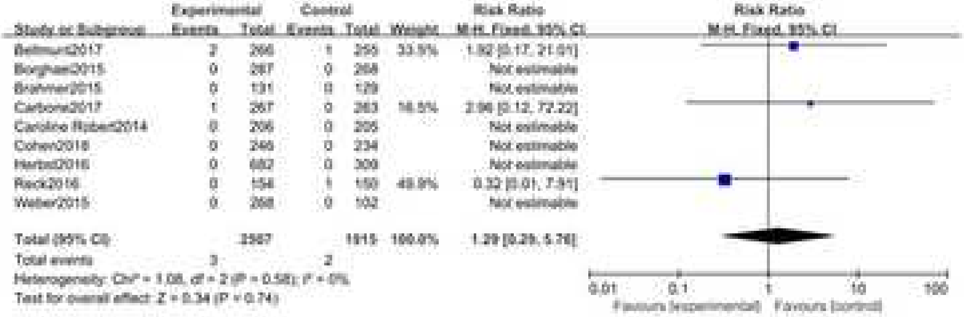

AKI g1-5

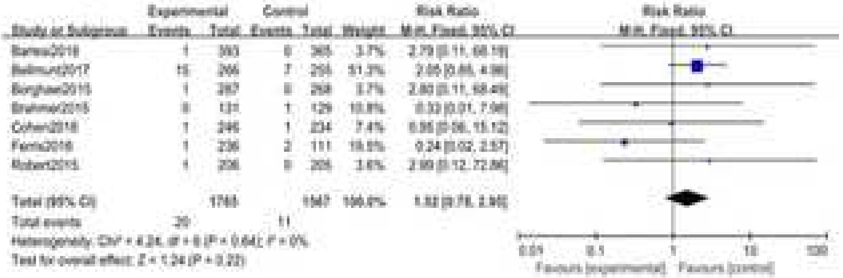

AKI g3-5

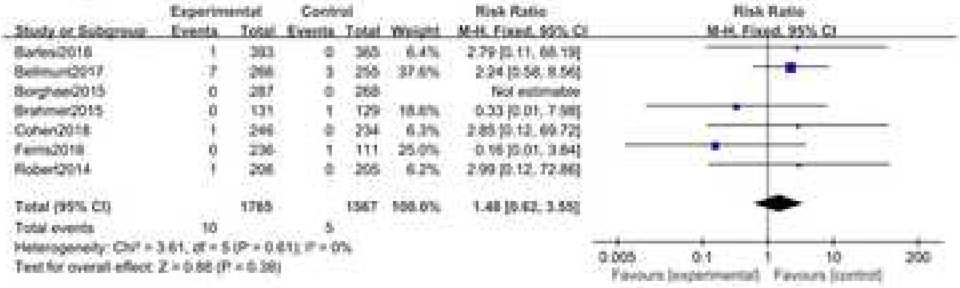

Nephritis g3-5

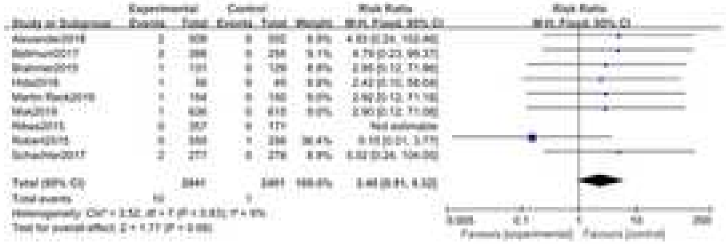

Increased blood creatinine g3-5

Study or Subgroup	Experimental Events	Experimental Total	Control Events	Control Total	Weight	Risk Ratio M-H, Fixed, 95% CI
Gandhi2018	2	405	0	202	26.6%	2.50 [0.12, 51.83]
Hodi2016	1	94	0	46	26.7%	1.48 [0.06, 35.74]
Horn2018	0	198	0	196		Not estimable
Langer2016	0	59	0	62		Not estimable
West2019	2	473	0	232	26.7%	2.46 [0.12, 50.99]
Wolchok2017	1	313	0	311	20.0%	2.98 [0.12, 72.89]
Total (95% CI)		1542		1049	100.0%	2.31 [0.49, 10.85]
Total events	6		0			

Heterogeneity: Chi² = 0.10, df = 3 (P = 0.99); I² = 0%
Test for overall effect: Z = 1.06 (P = 0.29)

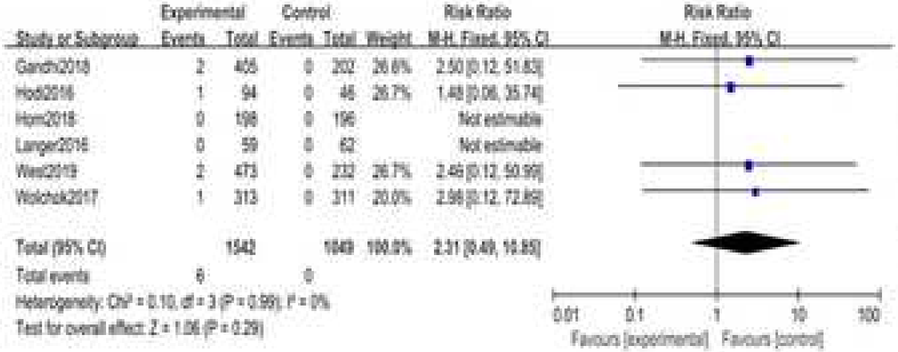

AKI g3-5

Study or Subgroup	Experimental Events	Experimental Total	Control Events	Control Total	Weight	Risk Ratio M-H, Fixed, 95% CI
Gandhi2018	8	405	1	202	28.6%	3.99 [0.50, 31.68]
Horn2018	2	198	0	196	10.8%	4.95 [0.24, 102.44]
Langer2016	2	59	0	62	10.5%	5.25 [0.26, 107.12]
Socinski2018	1	393	1	394	21.4%	1.00 [0.06, 15.97]
West2019	4	473	1	232	28.8%	1.96 [0.22, 17.45]
Total (95% CI)		1528		1086	100.0%	3.00 [1.01, 8.94]
Total events	17		3			

Heterogeneity: Chi² = 1.06, df = 4 (P = 0.90); I² = 0%
Test for overall effect: Z = 1.97 (P = 0.05)

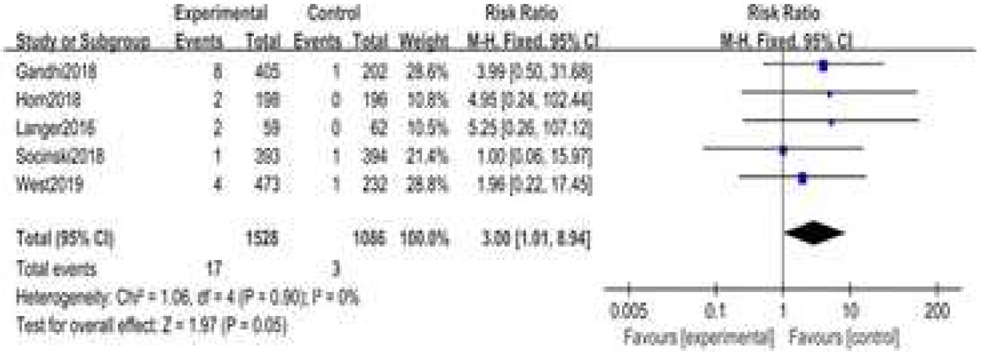

Nephritis g3-5

Study or Subgroup	Experimental Events	Experimental Total	Control Events	Control Total	Weight	Risk Ratio M-H, Fixed, 95% CI
Gandhi2018	6	405	0	202	15.4%	6.50 [0.37, 114.81]
Horn2018	1	198	0	196	11.6%	2.97 [0.12, 72.46]
Paz-Ares2018	2	278	2	280	46.0%	1.01 [0.14, 7.10]
Schmid2018	0	452	0	438		Not estimable
Socinski2018	1	393	0	394	11.5%	3.01 [0.12, 73.60]
West2019	1	473	0	232	15.5%	1.47 [0.06, 36.06]
Total (95% CI)		2199		1742	100.0%	2.38 [0.74, 7.68]
Total events	11		2			

Heterogeneity: Chi² = 1.34, df = 4 (P = 0.85); I² = 0%
Test for overall effect: Z = 1.46 (P = 0.15)

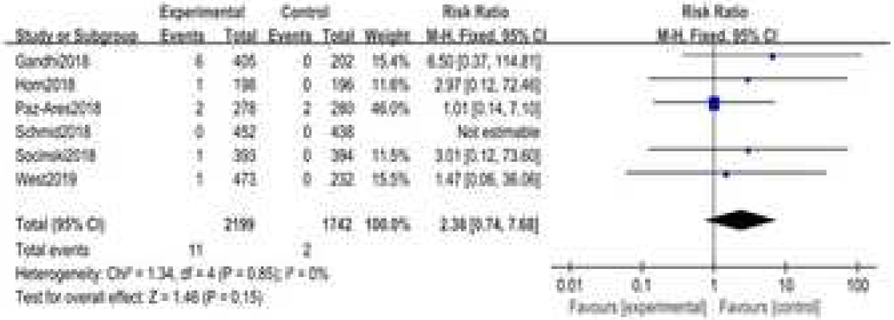

Table S1 The risk estimates that compared PD-1/PD-L1 inhibitors vs.chemotherapy.

AE type	RR (95%CL), P value	
	Grade1-5	Grade3-5
Increased blood creatinine	0.72(0.34,1.55),0.40	1.29(0.29,5.76),0.74
AKI	1.52(0.78,2.95),0.22	1.48(0.62,3.55),0.38
Nephritis	2.77(1.09,6.99),0.03	2.40(0.91,6.32),0.08
